# Bone Formation and Adaptive Morphology of the Anterior Tibial Muscle in 3-mm Daily Lengthening Using High-Fractional Automated Distraction and Osteosynthesis with the Ilizarov Apparatus Combined with Intramedullary Hydroxyapatite-Coated Wire

**DOI:** 10.1155/2019/3241263

**Published:** 2019-04-21

**Authors:** A. V. Popkov, N. A. Kononovich, G. N. Filimonova, E. N. Gorbach, D. A. Popkov

**Affiliations:** ^1^Laboratory for Limb Lengthening and Deformity Correction, Russian Ilizarov Scientific Center for Restorative Traumatology and Orthopedics, 6 M. Ulyanova Street, Kurgan, Russia; ^2^Experimental Laboratory, Russian Ilizarov Scientific Center for Restorative Traumatology and Orthopedics, 6 M. Ulyanova Street, Kurgan, Russia; ^3^Laboratory of Morphology, Russian Ilizarov Scientific Center for Restorative Traumatology and Orthopedics, 6 M. Ulyanova Street, Kurgan 640014, Russia; ^4^Neuroorthopedic Clinic, Russian Ilizarov Scientific Center for Restorative Traumatology and Orthopedics, 6 M. Ulyanova Street, Kurgan, Russia

## Abstract

**Purpose:**

We studied osteogenesis and morphofunctional features of the anterior tibial muscle using 3-mm high-frequency automated lengthening with the Ilizarov apparatus alone and in combination with intramedullary nailing.

**Material and Methods:**

Tibia was lengthened with a round-the-clock automated distractor at a 3-mm daily rate for 10 days in 16 mongrel dogs. In group 1 (n = 8), a 1.8-mm intramedullary titanium wire coated with hydroxyapatite was introduced into the tibial canal followed by Ilizarov frame mounting and transverse osteotomy of the diaphysis. Distraction mode was 0.025 mm x 120 increments a day. In group 2 (n = 8), distraction mode was the same but nailing was not used. Bone formation and the anterior tibial muscle were studied at two time points: (1) upon distraction completion; (2) three months after the apparatus removal. Bone formation was studied radiographically. Muscle preparations were examined histologically and stereomicroscopically.

**Results:**

There was a threefold reduction in the distraction time in both groups. Consolidation took 13.83±4.02 days in group 1 and 33.7±2.4 days in group 2. Muscle macropreparations of the experimental limb in group 1 at study time points did not show significant differences from intact tissues. Muscle histostructure in both groups was characterized by activation of angiogenesis and myohistogenesis, but the volumetric density of microvessels in the lengthening phase was three times higher in group 1.

**Conclusion:**

Combined technology significantly reduces the total lengthening procedure and does not compromise limb functions. Intramedullary HA-coated wires promote faster bone formation. The muscle was able to exhibit structural adaptation and plasticity of a restitution type.

## 1. Introduction

Automated high-fractional distraction with a daily rate of 1 mm in 60 steps was proposed by G.A. Ilizarov which later was under thorough investigation experimentally and tested in clinical settings by the team of his followers [1, 2]. It was found that high-frequency distraction is more beneficial for soft tissues because fewer alterations in nerves, cartilage, muscles, and functions were revealed [3, 4].

However, automated distraction has not received a wide application due to several shortcomings. One of them was that the same classical daily rate of 1 mm was achieved with automated distracters [5]. Therefore, the period of total external fixation osteosynthesis did not decrease. On the other hand, the cost of automated drivers for distraction was high. Nevertheless, the automated distraction has remained on the agenda [5, 6]. Our group of researchers started to investigate high speed high-fractional distraction modes experimentally to reveal how osteogenesis and other processes run under such conditions [7]. It was found that the increase in a daily rate up to 3 mm does not affect the activity of osteogenesis but results in a threefold decrease in the period of distraction phase. The next task was to shorten the distraction regenerate consolidation time.

Therefore, the idea to use hydroxyapatite-coated intramedullary titanium wires (HA-coated IMWs) for stimulation of osteogenesis and osteosynthesis time reduction was intensively studied [8, 9]. Bone tissue formation and osteointegration using regular distraction rate and combined osteosynthesis (Ilizarov frame and HA-coated IMWs) were reported, and the advantages of bone lengthening using a combined technique such as faster consolidation and fewer complications were shown [8–10].

Paraosseous tissues play an important role by limb lengthening through distraction osteogenesis. It was reported that high distraction frequency improved tissue adaptation during leg lengthening in an animal model and humans [3, 4]. Muscle histology was the same by distraction mode 1 mm/day in 1440 steps in comparison with 1 mm/day in 3 steps but resulted in better range of motion and somatosensory evoked potentials [3]. Recent research showed no difference in time to union or in the incidence of complications in comparison with manual low-frequent distraction [5]. The safety of automated distraction with the Ilizarov apparatus for bone and muscles was substantiated in the experimental works available in the Russian language sources, but the peculiarities of bone and muscle response to high speed automated bone distraction were not revealed to international readers [11].

Therefore, it was postulated that progressive distraction osteogenesis by using a 3-mm automated daily lengthening rate and Ilizarov apparatus osteosynthesis combined with HA-coated titanium IMW of the canine tibia would run faster and safer for the tibial muscles.

*Aim of Study*. We studied bone formation and morphological features of the anterior tibial muscle in the conditions of combined fixation (Ilizarov fixator + HA-coated intramedullary wire) and high-fractional 3-mm daily bone distraction and compared them with the findings of the same daily rate in the conditions of the Ilizarov fixation only.

## 2. Material and Methods

Lengthening of the tibia was performed with a round-the-clock automated distractor set at a daily rate of 3.0 mm for 10 days in 17 mongrel dogs of both sexes at the age of skeletal maturity weighing 15-20 kg. Intact group comprised 3 dogs.

**Figure 1:**
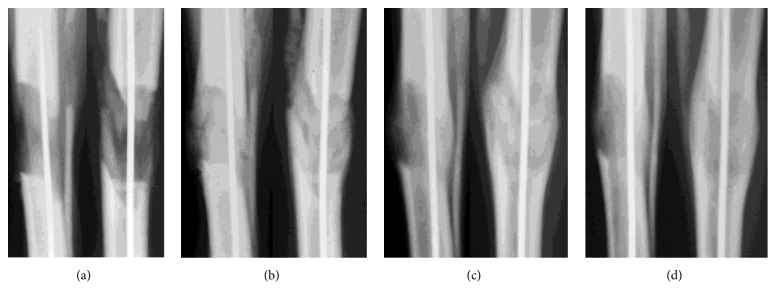
Lengthening site radiographs in group 1: (a) end of distraction; (b) end of fixation; (c) 30 days after frame removal; (d) 90 days after frame removal.

**Figure 2:**
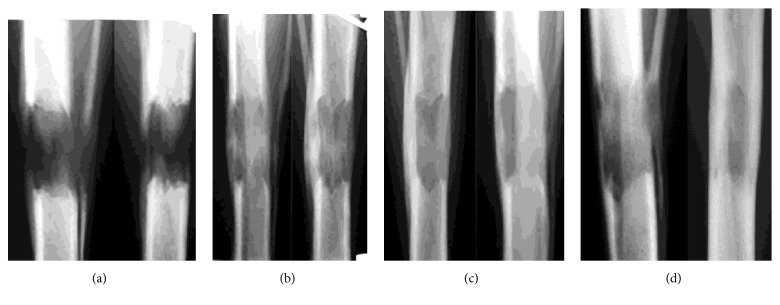
Lengthening site radiographs in group 2: (a) end of distraction; (b) end of fixation; (c) 30 days after frame removal; (d) 90 days after frame removal.

Under general anesthesia, 1.8-mm intramedullary wires made of a titanium alloy (Ti6Al4V) and coated with hydroxyapatite using MAO technology were introduced into the tibial canal in group 1 (n = 8). Osteosynthesis with an Ilizarov apparatus and transverse osteotomy of the tibia followed. Wires were not drilled through the anterior tibial muscle belly. Only three wires were used in each of the frame basic supports. The autodistractor mode was 0.025 mm x 120 increments (Figure 1). Distraction mode was the same but nailing was not used in group 2 (n = 8) which was a comparison group (Figure 2).

Distraction started seven days after transverse osteotomy of the tibia in both groups and continued 10 days to achieve about 3-cm lengthening or 14-16% of the initial tibial length. Radiography was performed in two standard views (AP and lateral) before osteosynthesis, during the intervention, on the day of distraction initiation, at the end of the elongation phase and fixation stages in the apparatus, and then on days 30 and 90 after the Ilizarov fixator removal.

The anterior tibial muscle samples were harvested upon distraction completion (4 dogs in each group) and 3 months after the removal of the apparatus (4 dogs in each group). The Ilizarov apparatus was dismantled after consolidation, when the X-ray images showed continuous periosteal response and regenerate middle zone was not distinguished or featured separate small portions. Clinical test (rotation and flexion loading) for absence of any mobility and pain was negative. The intramedullary wires were not removed in group 1.

General health of the animals and their limb functions, food and water consumption, and the presence or absence of neurological and infectious complications were additionally assessed at the stages of the experiment. Animals were kept in individual boxes, receiving the same standard and nutritionally balanced meals and clean drinking water.

For histological examination, an anterior tibial muscle fragment harvested from the site in the projection of the bone regenerate was fixed in a mixture of equal volumes of 2% glutaric and paraformaldehyde and then was soaked into paraffin. Part of the material was postfixed in 1.0% osmic acid and polymerized in epoxy resins. Paraffin sections were made on the microtome “Bromma-2218” LKB (Sweden), stained with hematoxylin and eosin according to Van Gieson and Masson. Semithin sections were obtained with Nova LKB ultrasound (Sweden), stained according to M. Ontell, and used for stereometry. The preparations were examined with a stereomicroscope AxioScope A1 supplied with a digital camera “AxioCam” (Carl Zeiss MicroImaging GmbH, Germany). In order to carry out a stereologic analysis of the muscle tissue, the PhotoFiltre program collected primary data, calculating the volumetric density (mm^3^/mm^3^) of muscle fibers (VVmf), microvessels (VVmv), and endomysium (VVend).

For a histochemical study, separate pieces measuring 5–7 mm^2^ were dissected from a designated area of the anterior tibial muscle, placed on a previously labeled filter paper, and immersed in liquid nitrogen. Transverse cryostat sections, 10 *μ*m thick, were then produced with a microtome cryostat MK-25TU64-1-856-78 (Russia) at a temperature of -200°C. Reaction to myosin adenosine triphosphatase (ATPase) identified types of muscle fibers and microvessels.

Muscle tissue fragments were impregnated with camphene and air-dried for SEM study. Next, they were mounted on metal substrates and sprayed with a current conducting layer in a vacuum ion sprayer IB6 (Japan). Fibrillar architectonics of the endomysium and perimysium were studied using a JSM-840 scanning electron microscope (Japan).

The findings were analyzed using nonparametric statistics in AtteStat 10.8.8 version for Microsoft Excel. The reliability of differences was determined with the Wilcoxon W-test for independent samples.

The experiments were carried out in accordance with the requirements of the* European Convention for the Protection of Vertebrates Used for Experimental and Other Scientific Purposes* (Strasbourg, 1986), in accordance with the principles of laboratory practice (NIH Publication no. 85-23, revised 1985), and were approved by the ethics board of our institution.

## 3. Results

The dogs were followed up for 90 days after the Ilizarov fixator removal. No changes in animals' general health or food and water consumption were observed during the study. There were no complications of an infectious or neurological nature. The weight-bearing function of the experimental limb was maintained until the end of the experiment. However, lameness of a resting type was observed in the predistraction period and in the stage of lengthening. We did not detect any foot equinus in group 1 throughout the experiment. In group 2, foot equinus was present in several cases but the animals actively used the limb for walking during the entire period of distraction osteosynthesis. After frame removal, ankle function was restored without foot equines.

Visual examination did not reveal any soft tissue changes in the limb of all experimental animals. There were no failures in the works of the automated distractor used.

Mean bone lengthening measured 29.32 ± 0.65 mm at the end of distraction. In group 1, the distraction regenerate strength allowed the removal of the Ilizarov apparatus after a mean period of 13.83 ± 4.02 days after completion of distraction when a clinical test showed an absent painful pathological mobility of bone fragments in the lengthening area (Figure 1). In group 2, the Ilizarov frame was taken off at a mean of 33.7±2.4 days (Figure 2). Ninety days after the dismantling of the apparatus, the axis of the tibia was correct in both groups.

Visual examination of anterior tibial muscle macropreparations found that the muscles of both extremities in group 1 did not differ considerably in the color and volume, being brightly crimson and equally massive. In group 2, the muscle of the experimental limb had a smaller volume and a pale pink color. The superficial fascia of the experimental tibial muscle in group 1 samples was more thin and transparent as compared to the fascia in the preparations of the muscle from group 2.

**Figure 3:**
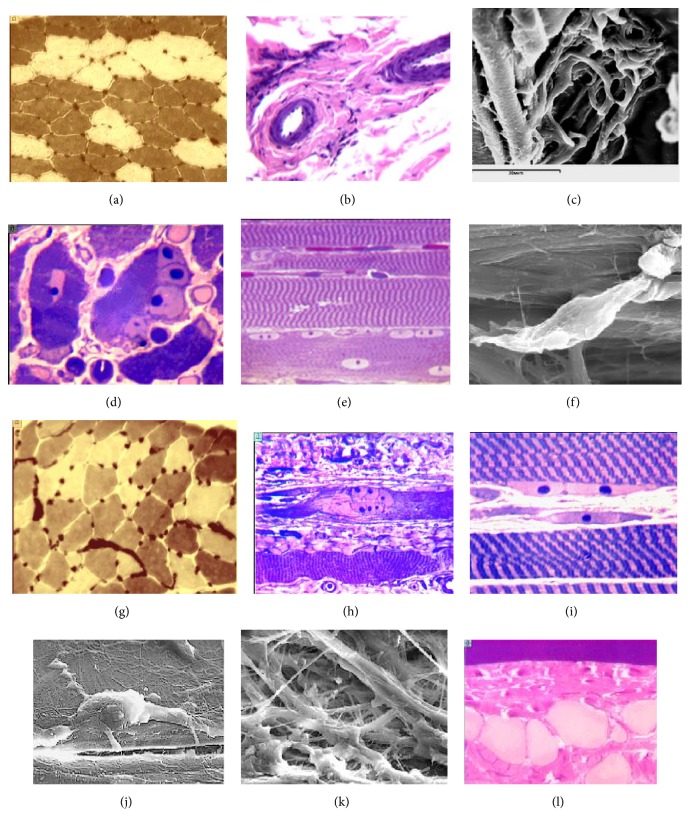
End of distraction. Muscle histostructure in group 1 (a, b, c, d, e, f) and group 2 (g, h, i, j, k, l): (a) metabolic fiber types. Polygonal profiles are preserved. Portion of white muscular fibers is increased (type 1). Sings of reinnervation (one type fibers grouping). Numerous capillaries. (b) Mild thickening of the adventitial layer in the perimysial vessels; (c) microvessels in the perimysium; (d) multinucleated myotube; (e) preserved transverse striation of muscle fibers; (f) spindle-shaped fibroblast in the endomysium; (g) polygonal type profiles of muscle fibers, microvessels filled with blood, signs of reinnervation; (h) regenerating muscle fiber in the center; (i) myoblast and spindle-shaped fibroblast in the endomysium; (j) active synthesizing fibroblast; (k) fibrillogenesis in the interstitial space; (l) adipocytes that replaced muscle fibers. Transverse cryostat sections, myosin ATPase reaction at pH 9.0 (Padykula and Herman). Magnification: 16x (lens), 12.5х (eyepiece) (a, g). Paraffin section stained with hematoxylin and eosin. Magnification: 40x (lens), 12.5x (eyepiece) (b). Semithin sections, stained by M. Ontell. Magnification: 100x (lens), 12.5x (eyepiece) (d, e, h, i, l). Electron scans. Magnification: 1600х (c, j); 2000x (f); 900x (k).

*At the end of distraction in group 1*, the histological analysis of the muscles revealed that most of the vision fields had a normal histostructure. Metabolic types of fibers were identified and their profiles retained polygonality and transverse striation (Figures 3(a) and 3(e)). Portion of white muscle fibers of type 1 was increased in the histological preparations with ATPase stain in comparison with the unstretched muscle. However, type 2 muscle fibers were greater in number than type 1 fibers (Figure 3(a)). There was some fiber type grouping indicating that reinnervation had occurred. The connective tissue layers were slightly enlarged as compared to the intact muscle. A marked fibrosis of the endo- and perimysium space was characteristic for some muscle fibers. A number of arterial vessels in these areas had a massive medium layer and increased adventitia (Figure 3(b)). Most perimysium vessels (81.7%) had a normal structure. Their number in the connective tissue sheath increased (Figure 3(c)). In the muscle fragments that featured an active plastic reorganization, the muscle fiber profiles lost their polygonality. Polymorphism of their diameters increased significantly. There were a lot of fine fibers, including the destroyed ones. The number of activated nuclei in the fibers increased (Figure 3(d)). Myoblasts, chains, and groups of nuclei were found (Figure 3(e)). The number of fibroblasts increased (Figure 3(f)). There were single contracted fibers and small labrocytes with tiny granules. The percentage of muscle fiber bundles with normal histological structure was 73.7% while fibrotic ones constituted 26.3%. Vessels with a closed lumen made 6.1%, and 12.2% of vessels had signs of adventitial fibrosis.

*In group 2*, muscle changes were similar but of greater severity. The portion of white muscle fibers (type 1) was insignificantly greater than that in group 1 (Figure 3(g)). There were more fibers that contracted to a varying degree. Regenerating muscle fibers were also detected (Figure 3(h)). Numerous active fibroblasts, oriented in the direction of the tension stress vector, were observed (Figures 3(i) and 3(j)). Fibrosis of the perimysium space caused by enhanced biosynthetic activity of fibroblasts was characterized by an increase in the volume of a fibrous connective tissue component (Figure 3(k)). Labrocytes were common and degenerated muscle fibers were present as well as the fields of adipocytes that had replaced muscle fibers (Figure 3(l)). The percentage of bundles of muscle fibers with preserved histostructure was 66.4%. The ones with fibrosis constituted 28.6% and 5% degenerating ones. Perimysium vessels of normal structure were 77.6%, 8.3% were with a closed lumen, and 14.1% of the vessels showed signs of adventitial fibrosis.

According to the stereometry data, by the end of the distraction, in group 1 the volumetric fraction of muscle fibers reduced by 5 to 7.5 % from the respective values in intact dogs (Table 1). The volume microvessels and endomysium increased significantly by 148% and 7% (in some fields of view up to 20%), respectively. In group 2, the volumetric density of microvessels and muscle fibers did not differ significantly from the intact values while that of the endomysium was significantly lower.

**Figure 4:**
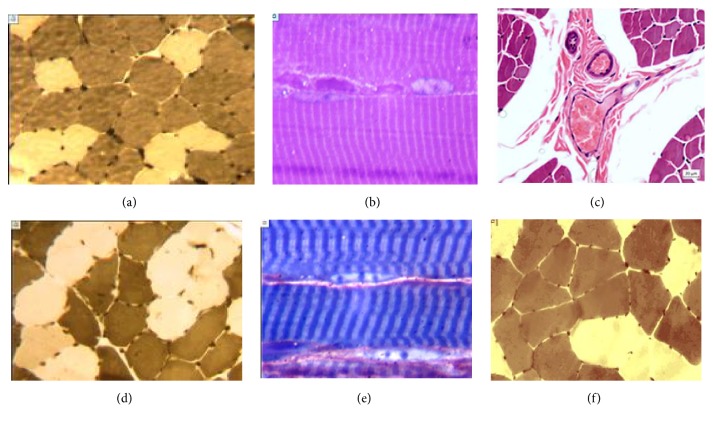
Ninety days after removal of the apparatus. Muscle histostructure in group 1 (a, b, c) and group 2 (d, e, f): (a) muscle structure approximated to normal; (b) normal striation of muscle fiber; (с) unchanged vessels of arterial and venous types in the intact perimysium; (d) variability of muscle fiber diameters, with increased portion of white fibers maintained (type 1); (e) preservation of increased number of fibroblasts in the endomysium; (f) normal muscle structure in an intact dog for comparison. Transverse cryostat sections, myosin ATPase reaction at pH 9.0 (Padykula and Herman). Magnification: 16x (lens), 12.5х (eyepiece) (a, d, f). Semithin sections, stained by M. Ontell. Magnification: 100x (lens), 12.5x (eyepiece) (b, e). Paraffin section, stained with hematoxylin and eosin; magnification: (с) 16x (lens), 12.5x (eyepiece).

*Three months after the removal of the apparatus, *the muscle structure of group 1 approximated the normal one and had polygonal fiber profiles. Their metabolic types were identified, and the variability of their diameters decreased relative to the distraction period. Inactivated nuclei were characteristic, and the portion of endomysium varied in different muscle fragments (Figures 4(a) and 4(b)).

In both groups, the perimysium vessels of the arterial and venous level were without any pathology like in intact dogs (Figure 4(c)). In group 2, the histostructural features of the anterior tibial muscle were similar to group 1 (Figures 4(d) and 4(e)), but adaptation structural changes still remained. In group 1, the volume fraction of microvessels significantly decreased (3-fold), but the portion of muscle fibers increased by 5-7.5% as compared with the end of distraction, which corresponded to group 2 and intact controls values. Regenerating fibers and paired myoblasts were visualized (Figure 4(e)). In both groups, the volume of endomysium was significantly lower as compared with the intact group (Figure 4(f)).

## 4. Discussion

It is universally accepted that a shorter time with the external fixator on is necessary to reduce the complications during the implementation of distraction osteogenesis such as pin-tract infection, pain, and muscle and joint stiffness that are associated with soft tissue tension [12]. The most common complications in tibial lengthening are foot equinus and contracture of the ankle joint [13, 14]. The mechanism of their formation is associated with the fact that an imbalance between the antagonist muscles develops in the course of tibial lengthening. The greatest resistance to stretching is shown by the gastrocnemius muscle belonging to the posterior group, in particular its distal tendon part (tendon calcaneus) [15]. The muscles of the anterior group are not loaded and as a result undergo greater changes. The dependence of the histostructural state and of the ability of any muscle to adapt to physical loading in animals of different ages was shown experimentally [16]. It was noted that by continuous training of stretch-shortening contractions of the posterior group of the lower leg muscles, atrophy develops in the functionally unloaded anterior tibial muscle [17]. Therefore, it seems obvious that the reduction of the distraction and fixation periods in tibial lengthening would decrease negative consequences associated with the imbalance. Our experiment enabled us to create favorable conditions for lengthening soft tissues because a sparing variant of the Ilizarov apparatus was applied when the wires did not enter the belly of the anterior tibial muscle, leaving it intact.

High distraction rate allowed for a 3-fold decrease in the distraction phase as compared with the classical rate of 1 mm per day. HA-coated intramedullary wires used in group 1 contributed to the strength of a newly formed diaphysis, induced osteogenic activity, and resulted in more than 2-fold decrease of the consolidation time with respect to group 2. In comparison with the classical lengthening, the period of hardware fixation after the cessation of distraction was reduced by more than 65% [18]. Coating of the wires used in our experiment is produced with the method of microarc oxidation (MAO) for better HA adhesion on the wire and good porosity for better osteointegration inside the medullary canal [9]. Our supposition of the effect of metal surface bioactive coatings to accelerate bone formation and osseointegration was confirmed again by comparing our groups.

The histological picture of the tibial muscle under the conditions of distraction in group 1 was characterized by the polygonality of the muscle fiber profiles in most fields of vision, which is characteristic of a functionally active muscle. It is known that muscles adapt well to distraction loads and the number of sarcomeres increases easily in order to maintain or restore the optimal length of the muscle [19]. The presence of sarcomerogenesis in bone lengthening was confirmed in many previous experimental studies [20–22].

In our work, the presence of visual fields with signs of active plastic reorganization when muscle fibers lost polygonality and numerous small fibers appeared, could indicate the emergence of regenerating and newly formed fibers. A large number of cells with euchromatic nuclei, independent or in groups, could be indirectly attributed to activated myosatellite cells of type II. Some authors showed that slight compression or cold would be enough to increase the number of myosatellitocytes which could be activated after training, with denervation or stretching [23].

Myosatellite cells of type II near the microvessels execute trophic supply to myocytes, participating in angiogenesis and myogenesis [24]. It was found that the satellite cells proliferated along the entire length of the anterior tibial muscles in the experiment with the use of 0.5-mm distraction for 2 steps daily in the rabbit's limb [25]. It is believed that distraction osteogenesis at a rate of 1.4 mm per day in skeletally immature animals leads to lengthening of the muscle due to sarcomerogenesis, forming a functionally active muscle [26].

The data of biochemical studies that had been already published also testify to the activation of myohistogenesis under the conditions of high-frequency automated distraction. They showed an increased role of the pentose-phosphate pathway, which is one of the basic molecular mechanisms providing repair processes in tissues [27]. Pentoses are used for the synthesis of nucleic acids. Skeletal muscle tissue metabolism reorganization in response to high-rate automated distraction testifies to the flexibility of the molecular mechanisms of muscle tissue, which ensures its preservation under given conditions. It was evidenced by the predominance of type 2 fibers as compared with type 1 at all stages of distraction osteosynthesis in both series, although in group 1 this difference was less pronounced. Therefore, we assume that there was no pathological disorder of tissue homeostasis.

The volumetric fraction of microvessels in group 1 was three times greater along with the increase in volumetric density of endomysium, similar to the process characteristic of growing puppies in the period of postnatal ontogenesis [28]. Probably, the HA-coated intramedullary titanium wire not only could promote a faster bone tissue regeneration and, accordingly, the functional activity even in the period of automated distraction, but also might have a beneficial effect on the proliferative activity of the soft tissue components adjacent to the distraction regenerate, in particular the tibial muscle. Calcium ions might diffuse from the hydroxyapatite layer into the surrounding tissues and the tone of vessels and blood flow may increase due to this diffusion [29].

Similar quantitative studies of muscle tissue in the available literature have not been found. In the present study, the activity of group I animals during the entire period of automated distraction and the functional load on the leg muscles resulted in the presence of the signs characteristic of the muscles of growing puppies with a significant increase in stromal elements in general (volume fraction of endomysium along with volume fraction of microvessels). Single small-sized labrocytes prove the absence of any inflammation in group 1 muscles. This probably explains why the macropreparations of the muscles of the tibia operated did not differ and even surpassed the muscle of the intact limb in volume, having a similar saturated color.

Thus, the functional state of the animals during the experiment, bone formation, and histostructural and stereometric characteristics of the anterior tibial muscle prove that the method of high-fractional automated bone distraction at a daily rate of 3 mm and the Ilizarov osteosynthesis reinforced with HA-coated IMWs has a stimulating effect on osteogenesis and accelerates structural recovery of the muscular tissue. Despite the fact that indirect signs of myohistogenesis were identified, the authors did not have the opportunity to use evidence-based immunohistochemical methods (unavailable) to detect regenerating fibers. It is a limitation of this study.

The anterior tibial muscle exhibited structural adaptation and plasticity of the restitution type. Nevertheless, further experiments and in-depth analysis of the morphofunctional characteristics of both muscular and paraosseous tissues are required. It is obvious that animal models cannot be directly extrapolated to humans. Nevertheless, new potential abilities of tissues, discovered in accelerated conditions of lengthening, could be promising factors for developing this technique for humans.

## 5. Conclusion

The technique of high-frequency automated bone distraction at a daily rate of 3 mm employing the Ilizarov osteosynthesis combined with HA-coated IMWs allows for significant reduction of the entire process of lengthening without compromising the functional activity of the limb.

## Figures and Tables

**Table 1:** Total volume of muscular fibers, microvessels, and endomysium in the anterior tibial muscle in the groups studied.

	End of distraction		90-day follow-up		Intact animals
Parameter	Group 1	Group 2	Group 1	Group 2	
VVmf, mm^3^/mm^3^	**0.775**±**0.011**	0.841±0.035	0.834±0.007	0.844±0.004	0.849 ±0.012

VVmv, mm^3^/mm^3^	**0.062±0.007**	0.023±0.004	0.027±0.003	0.022±0.002	0.025 ±0.002

VVend, mm^3^/mm^3^	**0.164±0.0101**	**0.136**±**0.004**	**0.14±0.006**	**0.133**±**0.002**	0.154 ±0.031

Values in bold are values of parameters that differ significantly from the corresponding parameters of the **intact dogs** (p≤0.05).

## Data Availability

The data used to support the findings of this study are available from the corresponding author upon request.
